# Regulation of T Lymphocyte Functions through Calcium Signaling Modulation by Nootkatone

**DOI:** 10.3390/ijms25105240

**Published:** 2024-05-11

**Authors:** Ji Min Lee, Jintae Kim, Su Jin Park, Joo Hyun Nam, Hyun Jong Kim, Woo Kyung Kim

**Affiliations:** 1 Department of Physiology, Dongguk University College of Medicine, 123 Dongdae-ro, Gyeongju 38066, Gyeongsangbuk-do, Republic of Korea; e_jimin@naver.com (J.M.L.); sujin900710@gmail.com (S.J.P.); jhnam@dongguk.ac.kr (J.H.N.); 2 Channelopathy Research Center (CRC), Dongguk University College of Medicine, 32 Dongguk-ro, Ilsan Dong-gu, Goyang 10326, Gyeonggi-do, Republic of Korea; jtmed92@gmail.com; 3 Department of Internal Medicine, Graduate School of Medicine, Dongguk University, 27 Dongguk-ro, Ilsan Dong-gu, Goyang 10326, Gyeonggi-do, Republic of Korea

**Keywords:** T lymphocyte, nootkatone, CRAC channel, Orai, K_V_1.3, K_Ca_3.1

## Abstract

Recent advancements in understanding the intricate molecular mechanisms underlying immunological responses have underscored the critical involvement of ion channels in regulating calcium influx, particularly in inflammation. Nootkatone, a natural sesquiterpenoid found in *Alpinia oxyphylla* and various citrus species, has gained attention for its diverse pharmacological properties, including anti-inflammatory effects. This study aimed to elucidate the potential of nootkatone in modulating ion channels associated with calcium signaling, particularly CRAC, K_V_1.3, and K_Ca_3.1 channels, which play pivotal roles in immune cell activation and proliferation. Using electrophysiological techniques, we demonstrated the inhibitory effects of nootkatone on CRAC, K_V_1.3, and K_Ca_3.1 channels in HEK293T cells overexpressing respective channel proteins. Nootkatone exhibited dose-dependent inhibition of channel currents, with IC_50_ values determined for each channel. Nootkatone treatment did not significantly affect cell viability, indicating its potential safety for therapeutic applications. Furthermore, we observed that nootkatone treatment attenuated calcium influx through activated CRAC channels and showed anti-proliferative effects, suggesting its role in regulating inflammatory T cell activation. These findings highlight the potential of nootkatone as a natural compound for modulating calcium signaling pathways by targeting related key ion channels and it holds promise as a novel therapeutic agent for inflammatory disorders.

## 1. Introduction

Understanding the significance of ion channels in regulating calcium influx is crucial for modulating immune responses, including inflammation. These channels play a key role in controlling intracellular calcium levels, which in turn directly influence the functions and responses of immune cells [1,2]. Upon activation by an antigen presenting cell (APC), immune cells undergo a process where phosphatidyl inositol 4,5-biphosphate (PIP_2_) becomes hydrolyzed and divided into diacylglycerol (DAG) and inositol 1,4,5-triphosphate (IP_3_). IP_3_ binds to the endoplasmic reticulum (ER) where the IP_3_ receptor (IP_3_R) is located, causing a Ca^2+^ ion stored in the endoplasmic reticulum (ER) to be released into the cytosol and depleting Ca^2+^ in the ER. This process triggers the accumulation of STIM1 (stromal interaction molecule 1) at ER–plasma membrane junctions, which in turn activates an ORAI1 pore unit, forming the Ca^2+^ release activated Ca^2+^(CRAC) channel and facilitating Ca^2+^ influx, a phenomenon known as store-operated calcium entry (SOCE) [3,4].

The influx of calcium through Ca^2+^ channels elevates cytosolic Ca^2+^ levels and depolarizes membrane potential. This initiates the activation of potassium channels like K_V_1.3 (voltage-gated potassium channel shaker-related subfamily member 1.3) and K_Ca_3.1 (potassium intermediate/small conductance calcium activated channel, subfamily N member 4) expressed in cell membranes, which are vital for restoring the resting membrane potential (RMP) by promoting K^+^ efflux. This regulatory mechanism ensures the sustained calcium signaling pivotal for optimal immune cell functionality [5,6]. These orchestrated processes mediate the downstream immune responses including immune cell activation, cytokine secretion and cell proliferation. Consequently, CRAC, K_V_1.3, and K_Ca_3.1 have emerged as important candidates for targeting immune reactions like autoimmune diseases or immunodeficiencies [7,8,9].

Considering the intertwined nature of inflammations, both of which are modulated by calcium signaling pathways, exploring the potential of natural compounds to regulate ion channels associated with Ca^2+^ influx holds promise for the development of novel anti-inflammatory or anti-allergic treatments. Nootkatone is a naturally occurring sesquiterpenoid found in *Alpinia oxyphylla* and various citrus species like grapefruit and is widely utilized in pharmacologic, food, and cosmetic industries. Nootkatone is known for exhibiting a broad spectrum of pharmacological properties including anti-inflammatory, anti-cancer, antioxidant, and anti-microbial effects. While the precise mechanism of action of nootkatone requires further elucidation through additional research, recent studies have demonstrated its ability, along with its natural sources, to modulate signal transduction pathways and regulate gene expression in diverse cell types [7,8,10]. Notably, it exhibits significant anti-inflammatory effects primarily mediated by signaling pathways, including the NF-κB pathway [11,12]. Given that the NF-κB pathway in immune cells like lymphocyte is also controlled by calcium signaling pathways [13,14], the pharmacological target of nootkatone could be linked with ion channel proteins.

Here, we provide an overview of the electrophysiological properties associated with ion channels that could be related to the pharmacological effects of nootkatone, including CRAC, K_V_1.3, and K_Ca_3.1 channels. This underscores the potential of nootkatone to play an important role in regulating calcium signaling in multiple ways. Additionally, we confirmed that T cell proliferation could be downregulated via nootkatone treatment without affecting the viability of cells through Ca^2+^ signal regulation. Therefore, this study suggests that nootkatone is a candidate natural molecular compound that offers potential for novel anti-inflammatory treatments.

## 2. Results

### 2.1. Calcium Transporting CRAC Channel Activation Is Regulated by Nootkatone

**Figure 1:**
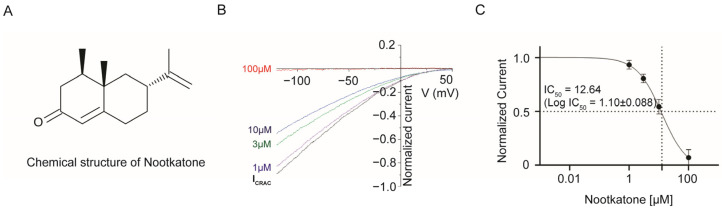
Nootkatone’s inhibitory effect on CRAC channel activity. Membrane currents were recorded via whole-cell voltage clamp. (**A**) Chemical structure of nootkatone. (**B**) Nootkatone (1, 3, 10, 100 µM) was applied after confirming the I_CRAC_ in hORAI overexpressed HEK293T cells. Representative current (I)–voltage (V) relationship curves of normal I_CRAC_ (black trace) and decreased current in 1, 3, 10, 100 µM concentrations of nootkatone are shown. (**C**) Relative amplitudes of normalized I_CRAC_ current are plotted and fitted to a dose-response curve. Data are presented as mean ± S.E.M (*n* = 4 or 5). Approximate half inhibition is achieved in 10 µM condition (R^2^ = 0.98, IC_50_ = 12.64).

Our investigation examined the impact of nootkatone on CRAC channel activity, crucial for the calcium transport essential to immune cell function. Using the whole-cell patch clamp technique, we recorded I_CRAC_ in an HEK293T cell transfected with hORAI plasmid [15]. Nootkatone was applied at various concentration in the extracellular solution to assess its inhibitory effect on the CRAC channel and I-V curves were traced in response to ramp pulse protocols (Figure 1A,B). Fitting the average inhibited currents to a dose-response curve showed that half-maximal inhibitory concentrations (IC50) of nootkatone on I_CRAC_ were about 12.64 µM (log(IC_50_) = 1.10 ± 0.088), and only 7.1% of the maximal current was observed at 100 µM (Figure 1C).

### 2.2. Potassium Channel K_Ca_3.1 and K_V_1.3 Activation Is Regulated by Nootkatone

**Figure 2:**
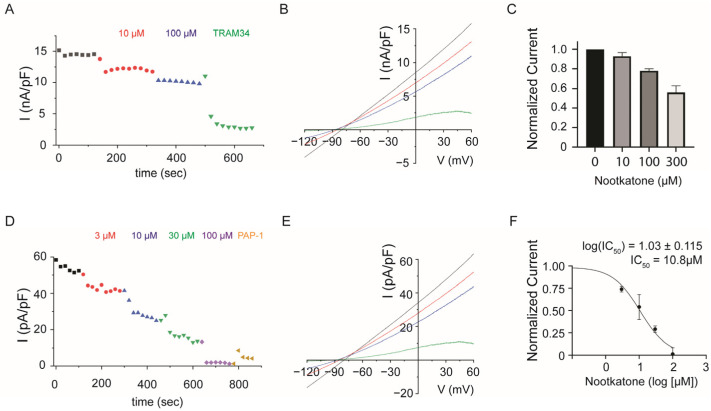
Nootkatone’s inhibitory effect on Potassium channel activity. (**A**) Representative chart trace of I_KCa3.1_ current inhibition caused by 10 and 100 µM concentrations of nootkatone. After confirming the channel activation, nootkatone solution was changed from lower to higher concentration. TRAM34 (Triarylmethane-34) is used as a potassium channel blocker to show the non-K^+^ current. (**B**) I-V relationship curve of representative trace. (**C**) Average value of each I_KCa3.1_ with nootkatone treatment was calculated by subtracting TRAM34 inhibited current. All data are expressed as the mean ± SEM (*n* = 3–5). (**D**) Representative chart trace of I_KV1.3_ current inhibition caused by 3, 10, 100 and 300 µM concentrations of nootkatone. After confirming the channel activation, nootkatone solution was changed from lower to higher concentration. PAP-1((5-(4-Phenoxybutoxy) psoralen) is used as a potassium channel K_V_1.3 blocker to show the non-K+ current. (**E**) I-V relationship curve of representative trace (timestamp of each curve is marked with arrow of same color in Figure 2D). (**F**) Average value of I_KV1.3_ was subtracted by PAP-1 inhibited current and normalized to show dose-response curve. Data are presented as mean ± S.E.M (*n* = 3–5, IC_50_ = 10.80, R^2^ = 0.96).

Next, we measured the K_Ca_3.1 channel current from the HEK293T cell line. Here, ramp pulses between −120 mV and 60 mV were given every 20 s and nootkatone was additionally treated with an extracellular bath solution during recording, and maximum current values for each ramp pulse were drawn (Figure 2A). After obtaining each I-V curve from 10 µM and 100 µM of nootkatone treatment, potassium channel inhibitor TRAM34 was applied and the remaining current was subtracted for normalization (Figure 2B). The average value of normalized nootkatone-inhibited K_Ca_3.1 channel currents shows that 100 µM of nootkatone could inhibit nearly 20% of the current, and we also found that 300 µM of nootkatone could additionally inhibit more than 40% of the current in additional experiments (Figure 2C).

We also measured the K_V_1.3 channel current in a Jurkat T cell and 3 µM, 10 µM, 30 µM, and 100 µM of nootkatone extracellular solution were subsequently given during whole-cell patch clamp recording (Figure 2D). Each I-V curve traced from different doses of nootkatone treatment is also represented in Figure 2E, and K_V_1.3 inhibitor PAP-1 was treated for further normalization process. When we fit these data into a dose-response curve, the log (IC_50_) value of the I_KV1.3_ current is calculated as 1.03 ± 0.115, and the IC50 as 10.8 µM (Figure 2F).

### 2.3. Store-Operated Calcium Entry Could Be Regulated by Nootkatone without Cytotoxicity

**Figure 3:**
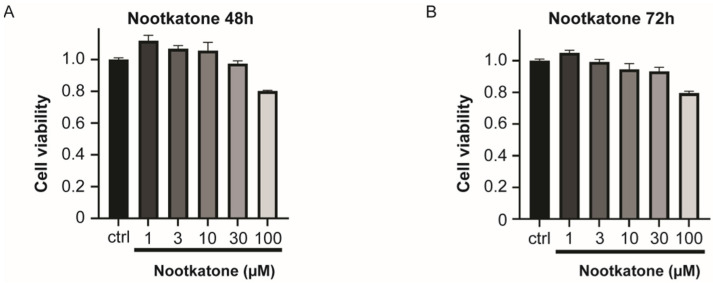
Cell viability was analyzed for 2~3 days after treatment with various concentrations of nootkatone in Jurkat T cells. For (**A**) 48 h or (**B**) 72 h after treatment, nootkatone showed no meaningful level of cytotoxicity, as the relative viability was not significantly lower than 80% of the total cell, compared with the control group. (*n* = 3).

We then conducted an experiment about the dose-dependent in vitro cytotoxicity of nootkatone. We treated various concentrations of nootkatone (1~100 µM) on Jurkat T cell and measured the viability of a cell after the next 48~72 h for each condition. Throughout the evaluation period, the viability of cells did not drop under 80%, indicating non-cytotoxicity (Figure 3).

**Figure 4:**
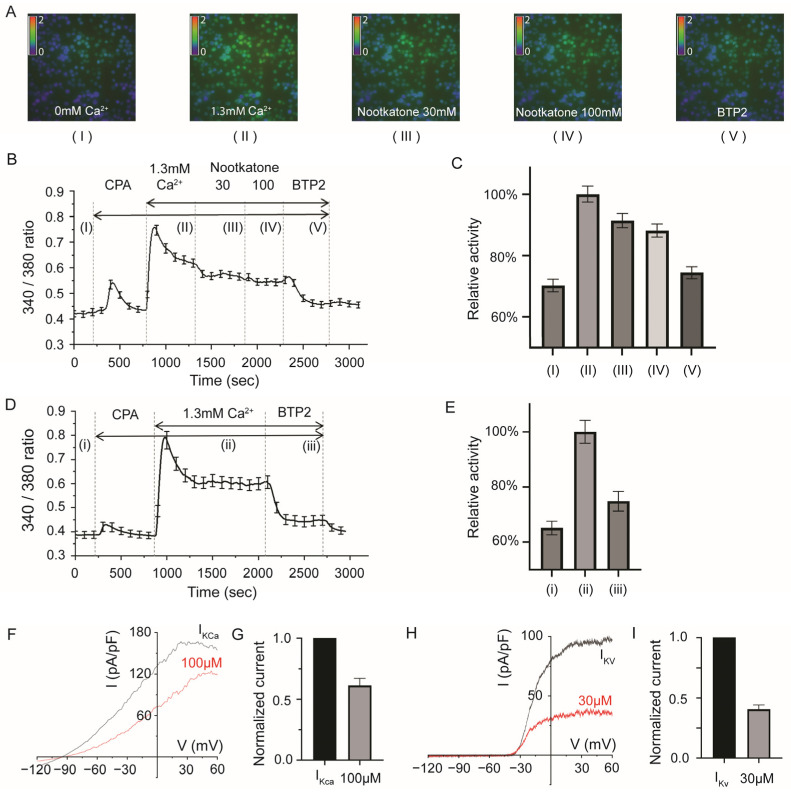
The inhibitory effect of nootkatone on store-operated Ca^2+^ entry (SOCE) was measured via Fura-2 calcium imaging. Activity was calculated with a 340/380 nm ratio, showing intracellular calcium ion concentration. (**A**) SOCE was induced via CPA (cyclopiazonic acid), with 1.3 mM of extracellular calcium starting calcium entry. Totals of 30 and 100 µM of nootkatone showed a lowering of 340/380 nm ratio; representative traces are shown. BTP2 was used to show the effect of the CRAC channel inhibitor. Representative images of Fura-2 calcium imaging are shown. Red and green signals indicate a higher level of calcium ion entry, while blue indicates lower SOCE activity. (I) Basal ratio, (II) CPA and 1.3 mM Ca^2+^ activated, (III) nootkatone 30 µM added, (IV) 100 µM added, and (V) BTP 10 µM added time points were used for image presentation and statistical comparison. Microscope magnification is 40× (**B**) Representative 340/380 ratio trace of calcium imaging. (**C**) Average normalized value of calcium signals in multiple cells in each timepoint, showing significant change after 30 and 100 µM of nootkatone treatment (the experiment was repeated three times, and the average number of cells is 54.3 ± 5.21, one-way ANOVA with bonferroni’s post-hoc test, *p* < 0.001). (**D**) Control 340/380 nm ratio trace of calcium imaging. (i) Basal ratio, (ii) CPA and 1.3 mM Ca^2+^ activated, (iii) BTP 10 µM added time points were used for statistical comparison. (**E**) Average normalized value of calcium signals in multiple cells in each timepoint (i)–(iii). (**F**) I-V relationship of K_Ca_ current in human primary CD4+ T cell. (**G**) Normalized value of K_Ca_ current. (**H**) I-V relationship of K_V_ current in human primary CD4+ T cell. (**I**) Normalized value of K_V_ current.

As we demonstrated the safe inhibition of multiple ion channels with 30~100 µM of nootkatone without inducing toxicity, we proceeded to investigate the inhibition ratio of Ca^2+^ inflow in a 100 µM nootkatone solution. In this experiment, cyclopiazonic acid (CPA), an ER calcium-depleting drug, was used to activate CRAC channels. Subsequently, 30 and 100 µM of nootkatone treatment was performed, revealing a significant reduction in the level of Ca^2+^ influx ([Ca^2+^]_i_) compared to the CRAC-activated cells without nootkatone treatment (Figure 4A–E). We also used human primary CD4+ T cells to show that K^+^ channels are effectively inhibited by nootkatone, similar to the result in Figure 2 (Figure 4F–I).

### 2.4. Nootkatone Inhibits T Lymphocyte Proliferation

**Figure 5:**
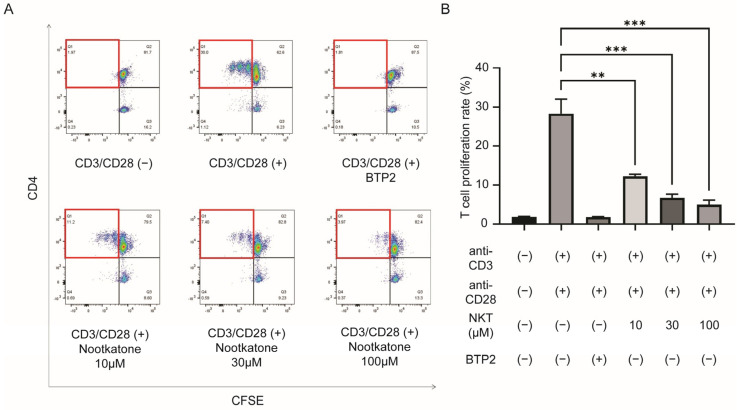
T cell proliferation analysis via flow cytometry (FACS). (**A**) In the upper panels, anti-CD3 and anti-CD28 untreated (left panel) and treated (middle panel) cell groups show an activation of T cell proliferation, and addition of BTP2 (CRAC channel inhibitor, right panel) showed inhibition of intracellular Ca^2+^ influx could minimalize T cell division. The cells within the red square are proliferating cells. In the lower panels, 10 (left), 30 (middle), and 100 (right panel) μM nootkatone treatment was additionally carried out in anti-CD3/CD28(+) T cells. Activated T cells showed partial inhibition of T cell proliferation, with dose-dependent efficacy. (**B**) T cell proliferation rate of each group. Statistical comparisons between anti-CD3/CD28(+) T cells and nootkatone treated groups were performed, and significant statistical differences were observed between groups (*n* = 3, one-way ANOVA with Dunnett’s post-hoc test, ** *p* < 0.01, *** *p* < 0.001).

We further investigated T cell proliferation rate reflecting immune cell activation. Anti-CD3 (5 µg/mL) and anti-CD28 (2 µg/mL) activated T cells were carboxyfluorescein diacetate succinimidyl ester (CFSE)-labeled and sorted through flow cytometry to find a proliferated ratio. Monitoring divided cells using diluted CFSE dye, anti-CD3 and anti-CD28 depleted cell groups and CRAC inhibitor BTP2 treated cell groups showed only 1.85 ± 0.097% and 1.77 ± 0.100% proliferated cell ratio, respectively, while 28.27 ± 3.739% proliferation rate was found in anti-CD3 and anti-CD28 antibody activated groups (Figure 5A). Compared with the control activated cell group, the nootkatone added group showed significantly lower proliferation rate, and only 4.94 ± 1.204% of proliferated cells were found in the 100 µM-treated group (Figure 5B,C).

In summary, nootkatone shows regulatory effects on calcium ion membranes transport via inhibition of CRAC and K^+^ channels. By affecting further signaling cascade, nootkatone could manage cell proliferation contributing to T cell mediated immune and inflammatory mechanisms.

## 3. Discussion

Nootkatone and related natural compounds are recognized for their anti-inflammatory effects, exhibiting therapeutic potential in conditions such as atopic dermatitis, mouse edema inflammation models, and septic inflammation models [16,17,18]. While the pharmacologic effects of nootkatone have been associated with the modulation of various different components of cell signaling pathways, little is known about the precise underlying mechanism, particularly in terms of cell electrophysiology [7]. Therefore, our study aimed to investigate the inhibitory effects of nootkatone on calcium-release activated calcium (CRAC) channels and potassium channels, K_Ca_3.1 and K_V_1.3. Our results also demonstrated a dose-dependent inhibition of channel activities by nootkatone.

The CRAC channel, formed by ORAI proteins within the cell membrane, is activated when the Ca^2+^ ion sensor STIM, located in the ER, detects internal Ca^2+^ levels and couples with it. This activation represents a primary mechanism for Ca^2+^ entry into non-excitable cells and plays a crucial role in regulating transcriptional processes essential for immune functions, particularly in T cell function [19,20,21]. This is evidenced by phenotype observations in patients or animal models with abnormal ORAI1 or STIM1 expression, which shows compromised CD4+ and CD8+ T cell functions related with humoral and cellular immunity, ultimately leading to immunodeficiencies and susceptibility to severe infections [19,22]. Considering that inflammation and autoimmune diseases are related with abnormal immune reactions, a CRAC channel inhibitor could be used to get rid of related diseases without causing severe side effects [23,24,25]. In our data, we showed that the CRAC current was successfully inhibited by treating nootkatone using patch clamp analysis (Figure 1).

In immune system functions, potassium channels are emerging as key modulators, especially for K_Ca_3.1 and K_V_1.3 channels cooperating to regulate several T cell functions [26]. Potassium channels play a significant role in Ca^2+^ signaling by maintaining membrane potential during cell activation. K_V_1.3 and K_Ca_3.1 channels are upregulated upon Ca^2+^ influx into cytosol during cell activation, and dysregulation of potassium channels may also contribute to immune disorders [5,27]. For example, K_V_1.3 channels are considered as therapeutic targets of autoimmune diseases like multiple sclerosis [28,29], while K_Ca_3.1 is implicated in various autoimmune diseases and inflammation developments, including rheumatoid arthritis, asthma, T-cell mediated colitis, and vascular inflammation [30,31,32,33]. Both potassium channels represent potential pharmacological targets in these diseases [6,9,26].

This is not only the case with T lymphocyte but also in a broader range of immune cells. In NK cells, for example, potassium channel K_Ca_3.1 also regulates cytotoxic activities by modulating Ca^2+^ ion entry [34], and K_V_1.3 is related to proliferation [35]. They cooperatively control immunologic functions such as anti-tumor activity. Also, in microglia, activation and proliferation is related with SOCE by CRAC and related potassium channels [36,37]. Although the complicated interplay between them needs further investigation, the relationship of those potassium channels and CRAC channels mediating inflammation could be promising in future drug development, such as targeting potassium channels for treating inflammation in microglia [38] or enhancing NK cell activity through a K_V_1.3 channel [35].

Our investigation reveals that nootkatone can inhibit the potassium channel current. We showed that 100 µM of nootkatone was sufficient to nearly block K_V_1.3 channel activity along with mild inhibition of K_Ca_3.1 channels (Figure 2 and Figure 4F,I). Given the compensatory effects of those potassium channels in regulating immune cell functions, nootkatone may provide an effective means of achieving dual inhibition of potassium ion flux for anti-inflammatory effects [26].

In conclusion, nootkatone could effectively interrupt the intracellular entry of store-operated Ca^2+^ currents, as confirmed using chemical indicator fura-2 (Figure 4). This inhibition is crucial for modulating downstream signaling pathways, such as NFAT, which are essential for various immunologic responses in T lymphocytes, including proliferation, differentiation, and cytokine production [2,13,39,40]. Additionally, our findings indicate that nootkatone reduces T cell proliferation rates in a dose-dependent manner, further supporting its potential anti-inflammatory effects.

However, it is also important to acknowledge the limitations of our study. We did not investigate the cytokine suppression effects of nootkatone, such as its impact on IL-2 production. Further exploration of its therapeutic in vivo effects is warranted to fully understand its potential clinical applications. Future research should focus on elucidating the precise mechanisms of action of nootkatone and exploring its therapeutic potential in various disease conditions. Nonetheless, our findings suggest that targeting calcium signaling using natural product-derived molecules like nootkatone could offer a promising alternative for the development of novel therapeutics for inflammation and allergic diseases. This approach could complement existing treatments, including corticosteroids and antihistamines, which are commonly used to inhibit the activation and proliferation of lymphocytes and mast cells but may have limitations in terms of efficacy or safety profiles [41]. Nootkatone holds promise as a therapeutic agent for patients requiring inhibition of unnecessary inflammatory activities without disrupting essential immune reactions.

## 4. Materials and Methods

### 4.1. Cell Culture

Human Embryonic Kidney 293 T (HEK293T) cell and Jurkat T cells were purchased from the American Type Culture Collection (ATCC, Manassas, VA, USA). HEK293T cells were maintained in a 37 °C, 10% CO_2_ incubator, using Dulbecco’s modified Eagle medium (DMEM, Welgene, Gyeongsan, Republic of Korea), supplemented with 10% fetal bovine serum (FBS, Welgene) and 1% penicillin/streptomycin (P/S; GE Healthcare, Chicago, IL, USA). Jurkat T cells were maintained in a 37 °C, 5% CO_2_ incubator, and the culture medium was RPMI1640 (Gibco, Waltham, MA, USA) with 10% FBS and 1% P/S.

### 4.2. Transient Transfection

HEK293T cells were co-transfected with human ORAI1 (hORAI1) and human STIM1 (hSTIM1) vectors to measure the CRAC current. Additionally, green fluorescence protein (pEGFP-N1, Life Technologies) was inserted to label the transfected cells. Transfection was conducted using Turbofect transfection reagent (Thermo Scientific, Waltham, MA, USA) following the manufacturer’s protocol. Details have been given previously [42].

### 4.3. Cell Cytotoxicity

Cell cytotoxicity was assessed using cell counting kit 8 (CCK-8). Sample preparation and analysis followed the protocol provided by the manufacturer. Jurkat T cells were seeded at a density of 2 × 10^4^ cells/well in 96-well plates, treated with nootkatone at concentrations of 1, 3, 10, 30, and 100 μM, and incubated for 72 h. Subsequently, 10 μL of CCK-8 solution was added to each well and incubated for an additional 3 h. Absorbance was then measured at 450 nm.

### 4.4. Electrophysiology

To measure the CRAC current, transiently transfected HEK293T cells were used, and the K_V_1.3 current in Jurkat T cells was measured directly. A stable cell line expressing K_Ca_3.1 in HEK293T (K_Ca_3.1—HEK) was used for measuring K_Ca_3.1 current (I_KCa3.1_). Currents were recorded using Axopatch 200B (Molecular Devices, Sunnyvale, CA, USA) and Digidata 1440A (Molecular Devices). More details have been reported previously [42]. The bath solution for recording the K_V_1.3 current (I_KV1.3_) and the K_Ca_3.1 current (I_KCa3.1_) contained 145 mM NaCl, 3.6 mM KCl, 10 mM 4-(2-hydroxyethyl)-1-piperazineethanesulfonic acid (HEPES), 5 mM glucose, 1.3 mM CaCl_2_, and 1 mM MgCl_2_, with pH adjusted to 7.4 using NaOH. The internal solution contained 5 mM NaCl, 140 mM KCl, 10 mM HEPES, 5 mM ethylene glycol-bis (β-aminoethyl ether)-N, N, N’, N’-tetraacetic acid, 2 mM Mg-ATP, 4.37 mM CaCl_2_, and 0.5 mM MgCl_2_ with pH adjusted to 7.2 using KOH. All chemicals were purchased from Sigma-Aldrich (St. Louis, MO, USA). Stock solutions were prepared in dimethyl sulfoxide (DMSO) and stored at −20 °C.

### 4.5. Fura-2 Ca^2+^ Imaging

The measurements of Ca^2+^ were performed with Jurkat T cells in a solution containing 145 mM NaCl, 3.6 mM KCl, 10 mM HEPES, 1 mM MgCl_2_, 1.3 mM CaCl_2_, 5 mM D-glucose, with the pH adjusted to 7.4 using NaOH. Jurkat T cells were incubated with Fura-2 AM (Thermo Fisher Scientific) at a final concentration of 1 μM for 30 min at 37 °C. These cells were then loaded onto 14 mm cover glass pre-coated with poly-L-Lysine. The ORAI1 channels were activated through store depletion induced by exposing cells to 30 µM of cyclopiazonic acid (CPA). Fura-2 AM fluorescence was excited at wavelengths of 340 nm and 380 nm and collected at an emission wavelength of 510 nm. Fluorescence signals were measured with a digital system including illuminator (pE-340 fura; CoolLED, Andover, UK) and camera (sCMOS pco.edge 4.2; PCO, Kelheim, Germany). The 340/380 ratio was obtained every 10 s and analyzed using NIS-Element AR Version 5.00.00 (Nikon, Tokyo, Japan).

### 4.6. Human Primary CD4+ T Lymphocyte Isolation

All procedures using human blood were approved by the Institutional Review Board (IRB), Dongguk University College of Medicine (2017-07-003 IRB). Human peripheral blood samples were obtained from healthy voluntary donors. Peripheral blood mononuclear cells (PBMCs) were isolated using Ficoll-Paque Plus medium (GE Healthcare, Chicago, IL, USA) density gradient centrifugation. Human naïve T lymphocytes were isolated using a human primary CD4+ T cell isolation kit (Miltenyi Biotec, Bergisch Gladbach, Germany), according to the manufacturer’s protocol and previous research [42].

### 4.7. T Cell Proliferation Assay

To assess cellular proliferation effects of nootkatone, flow cytometry (FACS) sorting was used. Here, cells were labeled with carboxyfluorescein succinimidyl ester (CFSE) to calculate the proportion of proliferated cells, using fluorescent dye [43]. The positive control cells and nootkatone treated cells were co-stimulated with anti-human CD3 (5 µg/mL) and CD28 (2 µg/mL) antibodies. CFSE signal intensity of the negative control group without anti-CD3 and anti-CD28 stimulation was used to determine the level of CFSE in non-proliferated cells. Nootkatone (10~100 µM) or BTP2 was also treated during anti-CD3 and anti-CD28 stimulation, if needed. Analysis of the ratio of proliferated CD4 cells, compared with total CD4 cells were carried out according to previous research [42].

### 4.8. Statistical Analysis

All data here are presented as mean ± standard error of the mean (SEM). Comparisons of multiple groups were performed using a one-way ANOVA analysis followed by post-hoc analysis.

Electrophysiological current traces, and related statistics were presented using Origin 2021b (OriginLab Corporation, Northampton, MA, USA). All other statistical analyses were performed using GraphPad Prism 10 (GraphPad Software Inc., San Diego, CA, USA). All statistical *p* values are indicated as follows: * *p* < 0.05, ** *p* < 0.01, *** *p* < 0.001, **** *p* < 0.0001, with *p* value < 0.05 considered as statistically significant.

## 5. Conclusions

Our findings provide compelling evidence of nootkatone’s ability to modulate critical calcium signaling pathways implicated in immune responses, including CRAC channel, K_Ca_3.1, and K_V_1.3. By elucidating its impact on cellular viability and Ca^2+^ influx mechanisms, our study underscores the potential of nootkatone as a promising candidate for the development of anti-inflammatory therapeutics.

## Data Availability

The data that support this study are available from the corresponding author upon reasonable request.
